# Pathophysiology of Vascular Remodeling in Hypertension

**DOI:** 10.1155/2013/808353

**Published:** 2013-07-22

**Authors:** Nicolás F. Renna, Natalia de las Heras, Roberto M. Miatello

**Affiliations:** ^1^Department of Pathology, School of Medicine, National University of Cuyo, Avenida Libertador 80, Centro Universitario, 5500 Mendoza, Argentina; ^2^Institute of Experimental Medicine and Biology of Cuyo (IMBECU), CONICET, Mendoza, Argentina; ^3^Department of Physiology, School of Medicine, Universidad Complutense, Plaza de Ramón y Cajal s.n., 28040 Madrid, Spain

## Abstract

Vascular remodeling refers to alterations in the structure of resistance vessels contributing to elevated systemic vascular resistance in hypertension. We start with some historical aspects, underscoring the importance of Glagov's contribution. We then move to some basic concepts on the biomechanics of blood vessels and explain the definitions proposed by Mulvany for specific forms of remodeling, especially inward eutrophic and inward hypertrophic. The available evidence for the existence of remodeled resistance vessels in hypertension comes next, with relatively more weight given to human, in comparison with animal data. Mechanisms are discussed. The impact of antihypertensive drug treatment on remodeling is described, again with emphasis on human data. Some details are given on the three mechanisms to date which point to remodeling resistance arteries as an independent predictor of cardiovascular risk in hypertensive patients. We terminate by considering the potential role of remodeling in the pathogenesis of endorgan damage and in the perpetuation of hypertension.

## 1. Introduction

In 1987, Glagov et al. reported the surprising finding that atherosclerotic narrowing of the arterial lumen is not simply the result of the enlargement of atherosclerotic lesions [1]. They described that the arteries, instead of remodeling the narrowed lumen, undergo many changes, such as increasing the outside diameter, to preserve blood flow.

This adaptability of the arteries is essential in arterial diseases. As with atherosclerotic coronary disease, peripheral vascular disease and hypertension may be considered a failure of the arterial wall to maintain a suitable mesh size to allow normal blood flow.

Recently, it has been suggested that the inability to remodel vessels properly is a way of “vascular insufficiency,” similar to that observed in the heart during heart failure. A definition of failure must begin with a description of the normal mechanisms that allow the artery walls to adapt to physiological requirements.

Hypertension elicits two different kinds of diffuse structural changes in the systemic microcirculation. One, termed rarefaction, consists in an abnormally low spatial density of arterioles, capillaries, and possibly venules. The other concerns structural modifications of resistance small arteries and arterioles, which lead to a reduction in lumen diameter and are grouped under the generic name of remodeling. We have recently reviewed rarefaction in detail. The focus of the present paper is on remodeling which probably accounts for the major part of long-term elevation of systemic vascular resistance (SVR) in hypertensive patients [2–9].

## 2. Definition of Vascular Remodeling

The vascular wall is formed by endothelium cells, smooth muscle cells, and fibroblasts interacting to form an autocrine-paracrine complex. During vascularization, the vascular wall detects changes in the environment, integrates these intercellular communication signals, and, through the local production of mediators, influences vascular structure and function. Vascular remodeling is an active process of structural change that involves changes in at least four cellular processes: cell growth, cell death, cell migration, and the synthesis or degradation of extracellular matrix. Vascular remodeling is dependent on dynamic interactions between local growth factors, vasoactive substances, and hemodynamic stimuli and is an active process that occurs in response to long-standing changes in hemodynamic conditions; however, it may subsequently contribute to the pathophysiology of vascular diseases and circulatory disorders [10].

In the historical work cited previously, the increased Wof hypertensive resistance vessels were uniformly ascribed to a higher volume of wall material per unit length of vessel (increased wall cross-sectional area (CSA)) or “hypertrophy.” It was assumed that smooth muscle cells in resistance vessels behaved as did left ventricular myocytes in the face of the increased pressure load and that growth took place mainly on the luminal side, leading to a structural reduction of internal diameter. The term remodeling was first applied to resistance vessels by Baumbach and Heistad, based on observations made in pial arterioles from stroke-prone spontaneously hypertensive rats (SPSHRs), to indicate a structural rearrangement of existing wall material around a smaller lumen [11–13].

Mulvany proposed that vascular remodeling should encompass any change in diameter noted in a fully relaxed vessel, not explained by a change in transmural pressure or compliance, and therefore due to structural factors [14–16].

To be operational, the classification necessitates appropriate methods for the measurement of resistance vessels dimensions. This problem is much harder than would seem at first sight.

To meet the definition of remodeling given previously, the respective sizes of hypertensive and normotensive small arteries and arterioles must be compared with the influence of the following factors either removed or controlled for: (i) vascular tone, (ii) transmural pressure, and (iii) vessel compliance.

Obviously, none of these requirements would be met by geometrical measurements made on standard histological sections prepared without perfusion of the tissue sample (e.g., shrinking artefacts) [17, 18].

One widely used approach, possible with small arteries (100 and 300 mm), is to carry out geometrical measurements on dissected segments put in standardized conditions in vitro.

## 3. Classification of Vascular Remodeling

**Figure 1:**
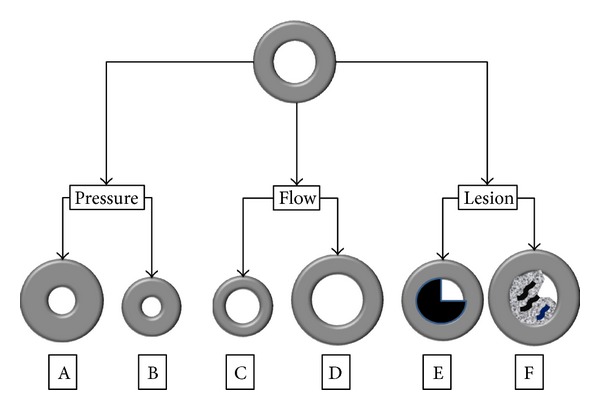
Changes are predominantly in media-to-lumen ratio (M/L), changing the vessel wall width for increased muscle mass (Figure 1(A)) or in the reorganization of cellular and noncellular elements (Figure 1(B)). Another mechanism of remodeling mainly involves changes in the dimensions of the lumen (Figures 1(C) and 1(D)). In this case, the restructuring of the active components and cell signals does not result in significant changes in the dimensions of the vascular lumen. Another form of vascular remodeling is microcirculation rarefaction (Figures 1(E) and 1(F)).

Consideration of morphological changes has changed over time. Feihl et al. [2] proposed a classification based on the response to increased blood pressure. These changes are displayed predominantly in media-to-lumen ratio (M/L), changing the vessel wall width for increased muscle mass (Figure 1(A)) or in the reorganization of cellular and noncellular elements (Figure 1(B)). These changes increase vascular reactivity, thus enhancing peripheral resistance. Another mechanism mainly involves changes in the dimensions of the lumen (Figures 1(C) and 1(D)). In this case, the restructuring of the active components and cell signals does not result in significant changes in the dimensions of the vascular lumen; the changes in vessel wall thickness are relatively small. Clinical examples of this type of restructuring include the dilation of vascular remodeling associated with a constantly high blood flow (Figure 1(D)) (e.g., arteriovenous fistula) or the loss of cellularity and extracellular matrix proteolysis, resulting in the formation of an aneurysm. Equally, a reduction in the diameter of the vascular mass results from a long-term reduction in blood flow (Figure 1(C)). In fact, microcirculation rarefaction (loss of the capillary zone) is another form of vascular remodeling that promotes hypertension and ischemic tissue. The architecture of the vascular wall is also markedly changed in response to vascular injury (Figures 1(E) and 1(F)). Neointima forms as part of the reparative response to injury, and its formation involves thrombosis, migration and vascular smooth muscle cells (VSMC) proliferation, matrix production, and the infiltration of inflammatory cells.

Hypertension is associated with structural changes in the resistance vessels such as reduction in lumen diameter and increase in M/L ratio. This mode of structural change has been called “remodeling” [19].

Structural changes in resistance vessels are described as a rearrangement process to understand the pathogenesis of the disease and its therapeutic approach. However, it has been discussed that the term “remodeling” is not ideal because it is frequently used to describe any change in the structure of the vessel or myocardium. To avoid this difficulty, some authors make four proposals [20].

First, the term “remodeling” is limited to situations where there is a change in the lumen of a relaxed vessel, as measured under standard intravascular pressure. The changes in the characteristics of the wall material do not take into account the change in the vascular lumen.

Second, the process of changing the vessel wall without changes in the amount or characteristics of the materials is termed eutrophic remodeling. This process can be characteristic of situations involving an increase in the amount of material (hypertrophic remodeling) and those involving a reduction in the amount of material (hypotrophic remodeling).

Third, changes associated with decrease or increase in lumen diameter should be classified as internal remodeling and external remodeling, respectively.

Finally, the remodeling process should be quantified. The term “remodeling index” refers to the variations of lumen referred to as eutrophic remodeling, depending on the changes in the wall section area.

The previous four proposals allow for accurate terminology. Thus, the increase in the M/L ratio and decrease in the lumen diameter in resistance vessels of patients with essential hypertension without any change in the amount of wall material are called inner eutrophic remodeling. The decrease in the lumen diameter of the renal afferent arteriole with a decrease in the amount of wall material is called inner hypotrophic remodeling.

Chronic changes in hemodynamic forces structurally alter the vascular wall. In addition, hemodynamic changes are not the only production mechanisms of vascular remodeling. The inflammatory response and changes in the components of the matrix have been suggested as important mediators in the vascular adaptation process [21].

**Figure 2:**
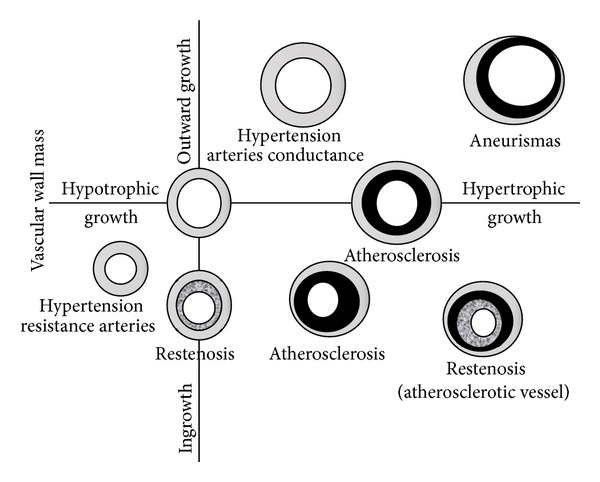
Schematic representation for the adaptation of these changes in different pathologies, including structural changes to the intima layer that contribute to remodeling of the vascular wall. Thus, outward remodeling compensates for atherosclerotic plaque growth and delays the progression of blood flow limitation during stenosis, whereas during restenosis, intimal hyperplasia causes a narrowing of the lumen.

Figure 2 highlights schematically the adaptation of these changes in different pathologies, including structural changes to the intima layer that contribute to remodeling of the vascular wall. Thus, outward remodeling compensates for atherosclerotic plaque growth and delays the progression of blood flow limitation during stenosis, whereas during restenosis, intimal hyperplasia causes a narrowing of the lumen.

In summary, vascular wall remodeling is the result of changes in cellular and noncellular components, depending on the disease process causing the changes. Changes in the growth and migration of VSMC, endothelial dysfunction, inflammatory processes, and the synthesis or degradation of extracellular matrix components may be present during the disease process.

## 4. Pathophysiology of Vascular Remodeling in Hypertension

### 4.1. Hypothesis of Inflammatory and Endothelial Dysfunction

The traditional view of atherosclerosis as a lipid storage disease is crumbling with growing evidence that inflammation is involved during all stages, from the initial injury to the final stage of thrombotic complications. The narrowing of the arterial lumen is not necessarily a sign of myocardial infarction, and treating narrowed blood vessels does not prolong life. Although invasive procedures are needed in some cases, we understand that medical treatment and lifestyle modification (diet and physical activity) produce benefits that may result from reductions in inflammatory processes [22].

Usually, endothelial cells (EC) prevent leukocyte adhesion. However, the triggers of atherosclerosis can initiate the expression of adhesion molecules on EC, mediating leukocyte adhesion to the arterial wall. A key part of this interaction is VCAM-1. It is likely that oxidized lipids can induce gene expression via the pathway initiated by the nuclear transcription factor *κ*B (NF-*κ*B), such as IL-1*β* and TNF-*α* [23].

This concept of vascular inflammatory disease allows a new approach for risk stratification and treatment. Increased levels of CAM are predictive of cardiac events and are an independent risk factor in men with coronary disease [24]. In our previous study, we demonstrated the presence of the endothelium as well as the products of NF-*κ*B signaling and VCAM-1 in an experimental model of metabolic syndrome in hypertensive rats receiving a fructose-rich diet (FFHR) [25].

Chemokines are low molecular weight cytokines responsible for mediating the maturation, differentiation, and migration of cells involved in the inflammatory response. In addition to this role, chemokines could promote reactive oxygen species (ROS) production and other cytokines during leukocyte infiltration of the vessel wall. Monocyte chemotactic protein-1 (MCP-1) is a chemokine that regulates the migration and infiltration of monocytes and macrophages into the site of inflammation. It is overexpressed in the presence of cardiovascular risk factors, especially in atherosclerotic lesions. Differential activation induces nuclear transcription factors such as NF-*κ*B and AP-1, which leads to the release of IL-6 and the proliferation of VSMC [26].

Cytokines are soluble proteins that form a complex signaling network critical in the regulation of innate and adaptive inflammatory response. Cytokines modulate the inflammatory response through their influence on the growth, development, and activation of leukocytes and other inflammatory cells. TNF-*α* is a key mediator in systemic inflammation with a significant role in the Th1 inflammatory pathway. The activity of TNF-*α* is varied and includes the production of interleukin CAM expression, cell migration and activation, and activation of metalloproteinases (MMP) and COX activity, promoting the procoagulant state. TNF-*α* is detected in endothelial cells and smooth muscle cells at all stages of the formation of atheromatous plaques [27].

There are over 30 members of the interleukin family. They are subdivided by the similar structure or homology of the receptor. The transformation from a vascular homeostasis inflammatory state is influenced by an imbalance between the proinflammatory and anti-inflammatory activities of interleukins. The role of IL-1 includes the stimulation of CAM, chemokines, growth factors, tissue factor, and other cytokines. The expression levels of the receptor antagonist IL-1Ra significantly increase in unstable angina compared with stable angina. Decreased levels of IL-1Ra after coronary stent placement may be linked to a low association with recurrent ischemia [28]. IL-6 is a multifunctional cytokine with a central role in inflammation. Elevated levels of IL-6 increase the risk of myocardial infarction and mortality in patients with coronary heart disease [29].

IL-10 has pleiotropic properties and influences different cell populations. Its most important role is in inflammatory vascular disease as part of the Th2 response. The expression of IL-10 decreases the expression of inflammatory cytokines, decreasing the Th1 phenotype. IL-10 also decreases NF-*κ*B signaling reducing synthesis of proinflammatory cytokines, CAM, chemoattractants, and growth factors [30, 31].

Endothelial dysfunction in FFHR causes an increase in the expression of NF-*κ*B and AP-1 and the posttranscriptional product VCAM-1. The expression of NF-*κ*B (p65) and AP-1 (c-fos) predominates throughout the vessel wall. Increased VCAM-1, as discussed in the literature, is a marker of vascular inflammation, vascular permeability, and endothelial dysfunction.

This experimental model produced an increased expression of several cytokines. This finding demonstrates that the vascular bed FFHR model presents a proinflammatory and proatherogenic microenvironment that favors vascular remodeling. C-reactive protein (CRP) was used to evaluate whether this local inflammatory process is also systemic and revealed significantly increased IL-6 expression in the liver. 

The potential importance of vascular wall inflammation as a therapeutic target remains an area not yet fully explored, where understanding the involvement of inflammatory mediators in vascular remodeling is relevant. The data suggest that oxidative stress and the subsequent activation of genes involved in the inflammatory process are actively involved in organ damage at the vascular level.

### 4.2. Vascular Remodeling and Extracellular Matrix Metalloproteinases

MMPs are tools for maintaining the homeostasis of extracellular structures. Their synthesis is induced by cytokines as well as cell-cell and cell-matrix interactions. Acute coronary syndromes are an example of an increase in clinical conditions, specifically in the vulnerable region of the plaque [32]. Exposure to oxidized low-density lipoproteins or TNF-*α* induces the expression of MT3-MMP, a protease that degrades atherosclerotic plaques and is expressed in macrophages [33, 34].

MMPs with accessory signaling molecules can modulate cell-cell interactions through the activation of signal transmission and release of cytokines and chemokines. By these effects, accessory signaling molecules can propagate the inflammatory response.

### 4.3. Vascular Remodeling and Acute Phase Reactants

The production of acute phase reactants is a normal physiological response to cytokine release in acute and chronic inflammatory conditions. Ultrasensitive quantification of CRP, when it is below the detection limits of the common assay, has a very important role in the detection of vascular inflammation and cardiovascular risk prediction. There is evidence that CRP is involved in atherosclerosis, especially during the early stages. It stimulates the production of proinflammatory cytokines in monocytes and macrophages [35] and mediates the expression of CAM, allowing for increased leukocyte adhesion and migration. Their increased expression suppresses endothelial nitric oxide synthase [36] and promotes a procoagulant state.

Multiple studies have determined that increases in CPR are an independent risk factor for developing atherosclerosis. Data from clinical studies indicate that this association is less important when viewed in healthy subjects and controls inflammatory markers such as IL-6 and fibrinogen [37, 38], whereas another study identified CRP as a predictor of diabetes mellitus independent of established risk factors. CRP also indicated a correlation with the risk of cardiovascular events in women with metabolic syndrome [39].

### 4.4. Vascular Remodeling and the Renin-Angiotensin-Aldosterone System

Another important pillar in the vascular remodeling process is the RAAS [40, 41]. To evaluate its participation, we studied the expression of AT1R and AT2R at the vascular level. In the experimental model of FFHR, we observed increased expression of AT1R and decreased expression of AT2R, promoting growth, vascular hypertrophy, and endothelial dysfunction. The release of ROS and initiation of vascular inflammation through different intracellular signaling cascades foster interconnections with other routes such as NAD(P)H oxidase and the growth factor receptor associated with insulin (IGFR).

**Figure 3:**
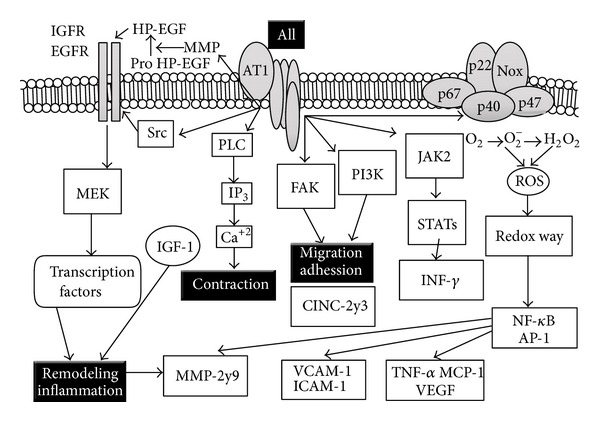
Associated intracellular cascades to physiopathology of vascular remodeling. In FFHR experimental model, the route associated with the satellite receptor and the IGFR subunit associated with NAD(P)H oxidase are the most important pathophysiological mechanisms. Also, the oxidative stress pathway stimulated by angiotensin activates redox-sensitive inflammatory molecules such as AP-1 and NF-*κ*B, which amplify vascular inflammatory response.

Figure 3 allows us to appreciate the AT1R-associated intracellular cascades. In this experimental model, the route associated with the satellite receptor and the IGFR subunit associated with NAD(P)H oxidase are the most important pathophysiological mechanisms. The FAK pathways PI3K and JAK2 generate stimuli and trigger contraction, migration, and cell adhesion via intranuclear promoters that synthesize ICAM-1 and VCAM-1. EGFR and IGFR amplified pathways are associated with cellular growth and hypertrophy as a result of insulinogenic stimuli and permit activation of collagenase, which modifies the extracellular matrix. Finally, the oxidative stress pathway stimulated by angiotensin activates redox-sensitive inflammatory molecules such as AP-1 and NF-*κ*B, which amplify the inflammatory response by cytokines, chemokines, and lymphokines to ultimately induce more vascular inflammation.

Angiotensin II is the main effector of the RAAS in the homeostatic regulation of the cardiovascular system and in the pathogenesis of cardiovascular disease. Aldosterone interacts with mineralocorticoid receptors (MR), causing endothelial dysfunction, facilitating thrombosis, reducing complacence, causing vascular hypertrophy and cardiac fibrosis and generating pathological remodeling. Aldosterone also induces the growth and proliferation of VSMC. A classical genomic action of aldosterone on MR is the translocation of this Aldo-MR complex into the nucleus, where it interacts with promoters to posttranscriptionally regulate gene and protein expression. For this path, increased Ki-ras2A expression (small and monomeric GTP-binding protein), which is associated with cardiac remodeling, generates fibrosis and cell proliferation by ERK1/2 possibly [42]. Recently, some authors have demonstrated that aldosterone stimulates EGFR intracellularly in CHO cells. The transactivation of this receptor has also been described as a crucial step in the cascade of MAPK signaling activated by angiotensin II. This pathway allows for “cross-talk” and mutual activation that allows the development of cardiovascular injury and subsequent remodeling. The latter route is via “fast” activation, which is different from genomic stimulation and stimulates MKP-1 and Ki-generated ras2A proliferation and vascular remodeling; this discovery explains the changes previously observed in other studies [43].

Noting the role of aldosterone in vascular remodeling in FFHR, we observed that chronic administration of spironolactone did not change the variables of metabolic syndrome that were partially reversed by oxidative stress. This can be explained by the relationship between aldosterone and the angiotensin II receptor AT1R, which sensitizes the effects and increased the postreceptor response [41].

In summary, abundant lines of evidence indicate the involvement of the RAAS in the pathophysiology of vascular remodeling; our observations in experimental pathology highlight the structural and functional changes.

In this special issue, different authors have tried to demonstrate the involvement of different pathophysiological mechanisms to clarify the vascular changes associated with hypertension and metabolic syndrome.

## 5. Clinical Data

The most feasible possibility for quantitative structural studies of resistance vessels in humans relies on the examination of small muscular (presumably resistance) arteries from biopsies of subcutaneous gluteal fat carried out under local anaesthesia. Small arteries can also be obtained from omental fat excised at the time of abdominal surgery [12, 44–47]. The dissected vessels are mounted in a wire or pressure myograph and characterized with the aforementioned methodology. Due to the invasive character of these procedures, most relevant studies are of modest size, typically involving between 10 and 20 subjects per group (with a few notable exceptions 49–51). Furthermore, untreated hypertensives are often patients in whom medication was withdrawn for a few weeks, rather than being newly diagnosed. 

In several studies, data indicate that small subcutaneous arteries of nondiabetic hypertensives undergo inward eutrophic remodeling. In contrast, it appears that diabetes on top of essential hypertension is associated with media hypertrophy, without a reduction of lumen diameter as measured in passive conditions. The same hypertrophy was also shown by one of these studies in normotensive diabetics, supporting a pressure-independent effect of diabetes on resistance vessel morphology.

Finally, the limited data available suggest that, contrary to the essential form, hypertension secondary to renovascular disease could promote media growth in human small subcutaneous arteries [48–51].

There are at least two caveats regarding the interpretation of these clinical data. First, the extent to which they might be contaminated by the aforementioned sampling problem is impossible to assess. Second, the subcutaneous vasculature is not necessarily representative of other vascular beds. There are a few observations to mitigate the latter concern. We may recall here the evidence of eutrophic remodeling in the intestinal microcirculation of hypertensive patients. In addition, a positive correlation has been found in hypertensive patients between coronary flow reserve and the M/L ratio of subcutaneous arteries, indeed supporting that hypertensive changes of microvascular structure were not limited to the subcutis [52, 53]. Finally, Harazny et al. [50] have very recently been able to evaluate the vascular remodeling of retinal arterioles in patients with treated hypertension and without advanced retinopathy (stage III or IV). To that effect, they used laser Doppler imaging whereby outer and inner diameters were, respectively, determined from reflection and perfusion images. Results indicated a higher ratio when blood pressure control was poorer than when it was satisfactory.
